# Time-Warp–Invariant Neuronal Processing

**DOI:** 10.1371/journal.pbio.1000141

**Published:** 2009-07-07

**Authors:** Robert Gütig, Haim Sompolinsky

**Affiliations:** 1 Racah Institute of Physics, Hebrew University, Jerusalem, Israel; 2 Interdisciplinary Center for Neural Computation, Hebrew University, Jerusalem, Israel; 3 Center for Brain Science, Harvard University, Cambridge, Massachusetts, United States of America; UC Berkeley, United States of America

## Abstract

Fluctuations in the temporal durations of sensory signals constitute a major source of variability within natural stimulus ensembles. The neuronal mechanisms through which sensory systems can stabilize perception against such fluctuations are largely unknown. An intriguing instantiation of such robustness occurs in human speech perception, which relies critically on temporal acoustic cues that are embedded in signals with highly variable duration. Across different instances of natural speech, auditory cues can undergo temporal warping that ranges from 2-fold compression to 2-fold dilation without significant perceptual impairment. Here, we report that time-warp–invariant neuronal processing can be subserved by the shunting action of synaptic conductances that automatically rescales the effective integration time of postsynaptic neurons. We propose a novel spike-based learning rule for synaptic conductances that adjusts the degree of synaptic shunting to the temporal processing requirements of a given task. Applying this general biophysical mechanism to the example of speech processing, we propose a neuronal network model for time-warp–invariant word discrimination and demonstrate its excellent performance on a standard benchmark speech-recognition task. Our results demonstrate the important functional role of synaptic conductances in spike-based neuronal information processing and learning. The biophysics of temporal integration at neuronal membranes can endow sensory pathways with powerful time-warp–invariant computational capabilities.

## Abstract 2

A biophysical mechanism acting in auditory neurons allows the brain to process the high variability of speaking rates in natural speech in a time-warp-invariant manner.

## Author Summary

The brain has a robust ability to process sensory stimuli, even when those stimuli are warped in time. The most prominent example of such perceptual robustness occurs in speech communication. Rates of speech can be highly variable both within and across speakers, yet our perceptions of words remain stable. The neuronal mechanisms that subserve invariance to time warping without compromising our ability to discriminate between fine temporal cues have puzzled neuroscientists for several decades. Here, we describe a cellular process whereby auditory neurons recalibrate, on the fly, their perceptual clocks and allows them effectively to correct for temporal fluctuations in the rate of incoming sensory events. We demonstrate that this basic biophysical mechanism allows simple neural architectures to solve a standard benchmark speech-recognition task with near perfect performance. This proposed mechanism for time-warp–invariant neural processing leads to novel hypotheses about the origin of speech perception pathologies.

## Introduction

**Figure 1:**
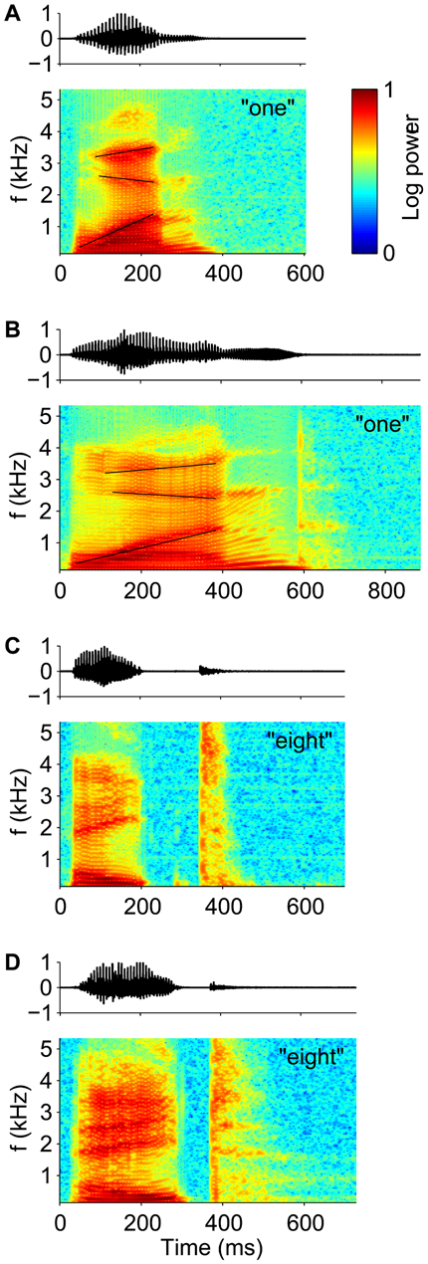
Time warp in natural speech. Sound pressure waveforms (upper panels, arbitrary units) and spectrograms (lower panels, color-code scaled between the minimum and maximum log power) of speech samples from the TI46 Word corpus [24], spoken by different male speakers. (A and B) Utterances of the word “one.” Thin black lines highlight the transients of the second, third, and fourth (bottom to top) spectral peaks (formants). The lines in (A) are compressed relative to (B) by a common factor of 0.53. (C and D) Utterances of the word “eight.”

Robustness of neuronal information processing to temporal warping of natural stimuli poses a difficult computational challenge to the brain [1]–[9]. This is particularly true for auditory stimuli, which often carry perceptually relevant information in fine differences between temporal cues [10],[11]. For instance in speech, perceptual discriminations between consonants often rely on differences in voice onset times, burst durations, or durations of spectral transitions [12],[13]. A striking feature of human performance on such tasks is that it is resilient to a large temporal variability in the absolute timing of these cues. Specifically, changes in speaking rate in ongoing natural speech introduce temporal warping of the acoustic signal on a scale of hundreds of milliseconds, encompassing temporal distortions of acoustic cues that range from 2-fold compression to 2-fold dilation [14],[15]. Figure 1 shows examples of time warp in natural speech. The utterance of the word “one” in (A) is compressed by nearly a factor of one-half relative to the utterance shown in (B), causing a concomitant compression in the duration of prominent spectral features, such as the transitions of the peaks in the frequency spectra. Notably, the pattern of temporal warping in speech can vary within a single utterance on a scale of hundreds of milliseconds. For example, the local time warp of the word “eight” in (C) relative to (D), reverses from compression in the initial and final segments to strong dilation of the gap between them. Although it has long been demonstrated that speech perception in humans normalizes durations of temporal cues to the rate of speech [2],[16]–[18], the neural mechanisms underlying this perceptual constancy have remained mysterious.

A general solution of the time-warp problem is to undo stimulus rate variations by comodulating the internal “perceptual” clock of a sensory processing system. This clock should run slowly when the rate of the incoming signal is low and embedded temporal cues are dilated, but accelerate when the rate is fast and the temporal cues are compressed. Here, we propose a neural implementation of this solution, exploiting a basic biophysical property of synaptic inputs, namely, that in addition to charging the postsynaptic neuronal membrane, synaptic conductances modulate its effective time constant. To utilize this mechanism for time-warp robust information processing in the context of a particular perceptual task, synaptic peak conductances at the site of temporal cue integration need to be adjusted to match the range of incoming spike rates. We show that such adjustments can be achieved by a novel conductance-based supervised learning rule. We first demonstrate the computational power of the proposed mechanism by testing our neuron model on a synthetic instantiation of a generic time-warp–invariant neuronal computation, namely, time-warp–invariant classification of random spike latency patterns. We then present a novel neuronal network model for word recognition and show that it yields excellent performance on a benchmark speech-recognition task, comparable to that achieved by highly elaborate, biologically implausible state-of-the-art speech-recognition algorithms.

## Results

### Time Rescaling in Neuronal Circuits

Whereas the net current flow into a neuron is determined by the balance between excitatory and inhibitory synaptic inputs, both types of inputs increase the total synaptic conductance, which in turn modulates the effective integration time of the postsynaptic cell [19]–[21] (an effect known as synaptic shunting). Specifically, when the total synaptic conductance of a neuron is large relative to the resting conductance (leak) and is generated by linear summation of incoming synaptic events, the neuron's effective integration time scales inversely to the rate of inputs spikes. Hence, the shunting action of synaptic conductances can counter variations in afferent spike rates by automatically rescaling the effective integration time of the postsynaptic neuron.

We implement this mechanism in a leaky integrate-and-fire model neuron driven by *N* exponentially decaying synaptic conductances 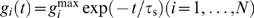. Here, 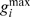 denotes the peak conductance of the *i*th synapse in units of *sec* ^−1^, and *τ*_s_ is the synaptic time constant. The total synaptic current, measured at rest, is given by
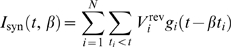
where 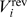 denotes the reversal potential of the *i*th synapse relative to resting potential and *t_i_* denote the arrival times of the spikes of the *i*th afferent. The factor *β* denotes a global scaling of all incoming spike times; *β* = 1 is the unwarped inputs. The total synaptic conductance, *G*_syn_(*t*,*β*), is
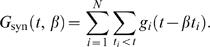



For fast synapses, the total synaptic current is essentially a train of pulses, each of which occurs at the time of an incoming spike and delivers a total charge of 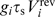. Changing the rate of the incoming spikes will induce a corresponding change in the timing of these pulses but not their charge. Therefore, ignoring the effect of time warp on the time scale of *τ*_s_, which is short relative to the time scale of voltage modulations, the total synaptic current obeys the following time-warp scaling relation, *I*_syn_(*βt*,*β*) = *β* ^−1^ *I*_syn_(*t*,1). A similar scaling relation holds for the total synaptic conductance. The evolution in time of the subthreshold voltage is given by
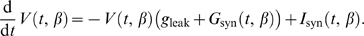
(1)


**Figure 2:**
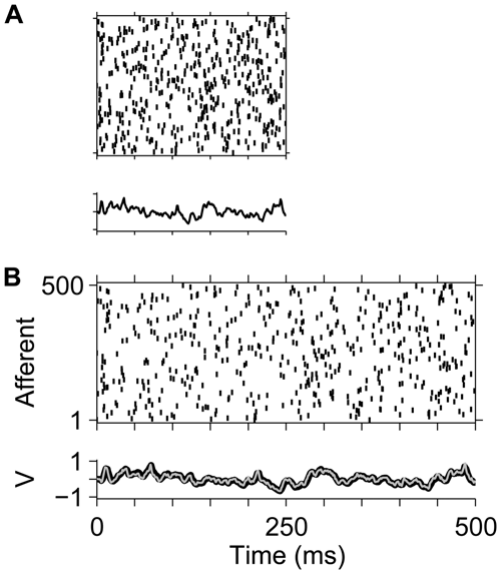
Time-warp–invariant voltage traces. Spike rasters show a random spike pattern across *N* = 500 afferents (*N*_ex_ = 250 excitatory and *N*_in_ = 250 inhibitory), each of which fires a single action potential at a random time chosen uniformly between 0 and 500 ms. Whereas the original spike pattern (*β* = 1) is shown in (B), the pattern displayed in (A) is compressed by a factor of *β* = 0.5. In each panel, the lower trace depicts the voltage *V*(*t*,*β*) induced by the spike patterns in our model neuron with balanced uniform synaptic peak conductances that resulted in a zero mean synaptic current at rest set to 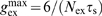 for excitatory synapses and 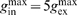 for inhibitory synapses. These values result in a mean total synaptic conductance of 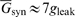. In (B), the voltage trace *V*(*t*,1) (thin grey line) is superimposed on the rescaled voltage trace *V*(*βt*,*β*) (thick black line) from (A).

Thus, *V* integrates the synaptic current with an effective time constant whose inverse is 1/*τ*_eff_ = *g*_leak_+*G*_syn_(*t*,*β*). If the contribution of *G*_syn_ is significantly larger than the leak conductance, then 1/τ_eff_ is rescaled by time-warp similar to *G*_syn_ and *I*_syn_, and, hence, the solution of Equation 1 is approximately time-warp invariant, namely, *V*(*βt*,*β*) = *V*(*t*,1). This result is illustrated in Figure 2, which compares the voltage traces induced by a random spike pattern for *β* = 1 and *β* = 0.5.

To perform time-warp–invariant tasks, peak synaptic conductances must be in the range of values appropriate for the statistics of the stimulus ensemble of the given task. To achieve this, we have devised a novel spike-based learning rule for synaptic conductances, the conductance-based tempotron. This model neuron learns to discriminate between two classes of spatiotemporal input spike patterns. The tempotron's classification rule requires it to fire at least one spike in response to each of its target stimuli but to remain silent when driven by a stimulus from the null class. Spike patterns from both classes are iteratively presented to the neuron, and peak synaptic conductances are modified after each error trial by an amount proportional to their contribution to the maximum value of the postsynaptic potential over time (see Materials and Methods). This contribution is sensitive to the time courses of the total conductance and voltage of the postsynaptic neuron. Therefore, the conductance-based tempotron learns to adjust, not only the magnitude of the synaptic inputs, but also its effective integration time to the statistics of the task at hand.

### Learning to Classify Time-Warped Latency Patterns

**Figure 3:**
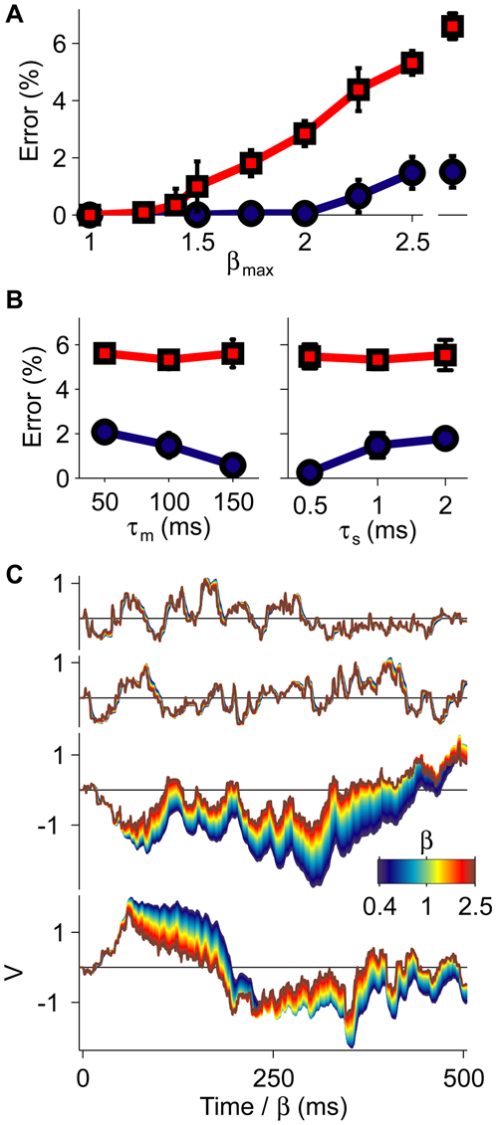
Classification of time-warped random latency patterns. (A) Error probabilities versus the scale of global time-warp *β*_max_ for the conductance-based (blue) and the current-based (red) neurons. Errors were averaged over 20 realizations, error bars depict ±1 standard deviation (s.d.). Isolated points on the right were obtained under dynamic time warp with *β*_max_ = 2.5 (see Materials and Methods). (B) Dependence of the error frequency at *β*_max_ = 2.5 on the resting membrane time constant *τ*_m_ (left) and the synaptic time constant *τ*_s_ (right). Colors and statistics as in (A). (C) Voltage traces of a conductance-based (top and second rows) and a current-based neuron (third and bottom rows). Each trace was computed under global time warp with a temporal scaling factor *β* (see Materials and Methods) (color bar) and plotted versus a common rescaled time axis. For each neuron model, the upper traces were elicited by a target and the lower traces by an untrained spike template.

We first quantified the time-warp robustness of the conductance-based tempotron on a synthetic discrimination task. We randomly assigned 1,250 spike pattern templates to target and null classes. The templates consisted of 500 afferents, each firing once at a fixed time chosen randomly from a uniform distribution between 0 and 500 ms. Upon each presentation during training and testing, the templates underwent global temporal warping by a random factor *β* ranging from compression by 1/*β*_max_ to dilation by *β*_max_ (see Materials and Methods). Consistent with the psychophysical range, *β*_max_ was varied between 1 and 2.5. Remarkably, with physiologically plausible parameters, the error frequency remained almost zero up to *β*_max_≈2 (Figure 3A, blue curve). Importantly, the performance of the conductance-based tempotron showed little change when the temporal warping applied to the spike templates was dynamic (see Materials and Methods) (Figure 3A). The time-warp robustness of the neural classification depends on the resting membrane time constant *τ*_m_ and the synaptic time constant *τ*_s_. Increases in *τ*_m_ or decreases in *τ*_s_ both enhance the dominance of shunting in governing the cell's effective time constant. As a result, the performance for *β*_max_ = 2.5 improved with increasing *τ*_m_ (Figure 3B, left) and decreasing *τ*_s_ (Figure 3B, right). The time-warp robustness of the conductance-based tempotron was also reflected in the shape of its subthreshold voltage traces (Figure 3C, top row) and generalized to novel spike templates with the same input statistics that were not used during training (Figure 3C, second row).

Synaptic conductances were crucial in generating the neuron's robustness to temporal warping. Athough an analogous neuron model with a fixed integration time, the current-based tempotron [22] (see Materials and Methods) also performed the task perfectly in the absence of time-warp (*β*_max_ = 1); its error frequency was sensitive even to modest temporal warping and deteriorated further when the applied time warp was dynamic (Figure 3A, red curve). Similarly, the voltage traces of this current-based neuron showed strong dependence on the degree of temporal warping applied to an input spike train (Figure 3C, bottom trace pair). Finally, the error frequency of the current-based neuron at *β*_max_ = 2.5 showed only negligible improvement upon varying the values of the membrane and synaptic time constants (Figure 3B), highlighting the limited capabilities of fixed neural kinetics to subserve time-warp–invariant spike-pattern classification.

Note that in the present classification task, the degree of time-warp robustness depends also on the learning load, i.e., number of patterns that have to classified by a neuron (unpublished data). A given degree of time warp translates into a finite range of distortions of the intracellular voltage traces. If these distortions remain smaller than the margins separating the neuronal firing threshold and the intracellular peak voltages, a neuron's classification will be time-warp invariant. Since the maximal possible margins increase with decreasing learning load, time-warp invariance can be traded for storage capacity. This tradeoff is governed by the susceptibility of the voltage traces to time warp. If the susceptibility is high, as in the current-based tempotron, robustness to time warp comes at the expense of a substantial reduction in storage capacity. If it is low, as in the conductance-based tempotron, time-warp invariance can be achieved even when operating close to the neuron's maximal storage capacity for unwarped patterns.

### Adaptive Plasticity Window

**Figure 4:**
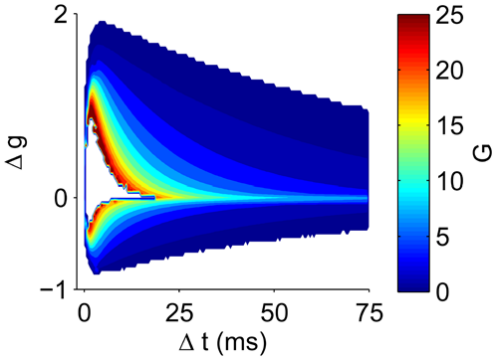
Adaptive learning kernel. Change in synaptic peak conductance Δ*g* versus the time difference Δ*t* between synaptic firing and the voltage maximum, as a function of the mean total synaptic conductance *G* during this interval (color bar). Data were collected during the initial 100 cycles of learning with *β*_max_ = 2.5 and averaged over 100 realizations.

In the conductance-based tempotron, synaptic conductances controlled, not only the effective integration time of the neuron, but also the temporal selectivity of the synaptic update during learning. The tempotron learning rule modifies only the efficacies of the synapses that were activated in a temporal window prior to the peak in the postsynaptic voltage trace. However, the width of this temporal plasticity window is not fixed but depends on the effective integration time of the postsynaptic neuron at the time of each synaptic update trial, which in turn varies with the input firing rate at each trial and the strength of the peak synaptic conductances at this stage of learning (Figure 4). During epochs of high conductance (warm colors), only synapses that fired shortly before the voltage maximum were appreciably modified. In contrast, when the membrane conductance was low (cool colors), the plasticity window was broad. The ability of the plasticity window to adjust to the effective time constant of the postsynaptic voltage is crucial for the success of the learning. As is evident from Figure 4, the membrane's effective time constant varies considerably during the learning epochs; hence, a plasticity rule that does not take this into account fails to credit appropriately the different synapses.

### Task Dependence of Learned Synaptic Conductance

**Figure 5:**
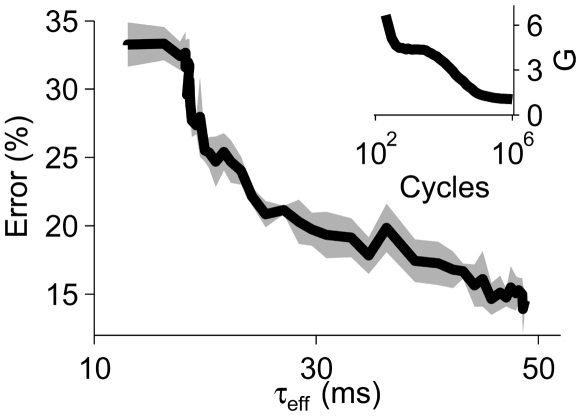
Task dependence of the learned total synaptic conductance. Error frequency of the conductance-based tempotron versus its effective integration time *τ*_eff_. After switching from time-warp to Gaussian spike jitter, *τ*_eff_ increased as the mean time-averaged total synaptic conductance *G* decreased with learning time (inset).

The evolution of synaptic peak conductances during learning was driven by task requirements. When we replaced the temporal warping of the spike templates by random Gaussian jitter [22] (see Materials and Methods), conductance-based tempotrons that had acquired high synaptic peak conductances during initial training on the time-warp task readjusted their synaptic peak conductances to low values (Figure 5, inset). The concomitant increase in their effective integration time constants from roughly 10 ms to 50 ms improved the neurons' ability to average out the temporal spike jitter and substantially enhanced their task performance (Figure 5).

### Neuronal Model of Word Recognition

**Figure 6:**
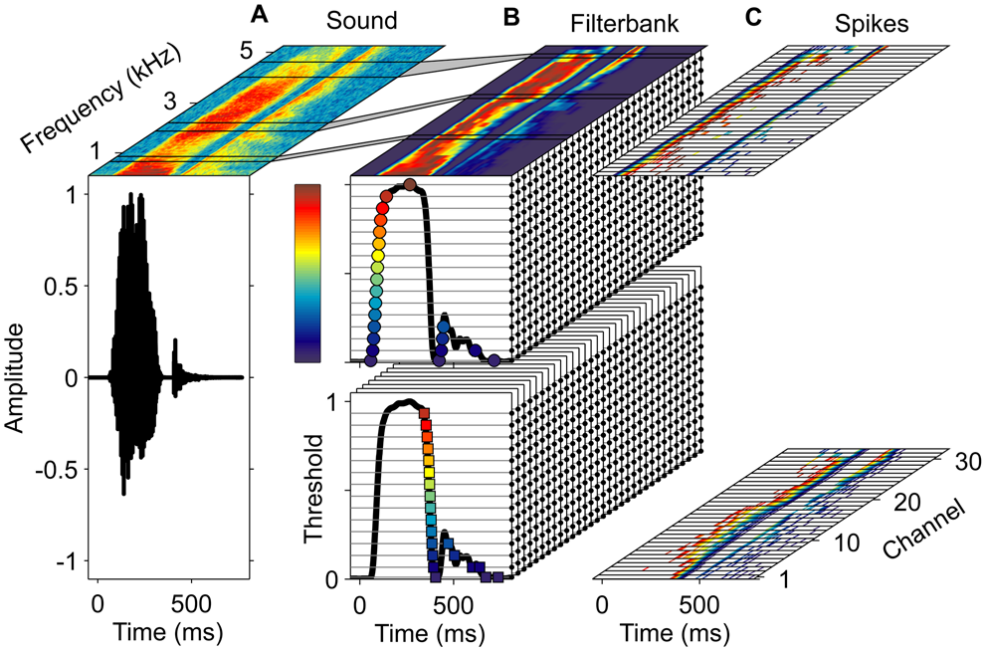
Auditory front end. (A and B) Incoming sound signal (bottom) and its spectrogram in linear scale (top) as in Figure 1D (A). Based on the spectrogram, the log signal power in 32 frequency channels (Mel scale, see Materials and Methods) is computed and normalized to unit peak amplitude in each channel ([B], top, colorbar). Black lines delineate filterbank channels 10, 20, and 30 and their respective support in the spectrogram (connected through grey areas). In each channel, spikes in 31 afferents (small black circles) are generated by 16 onset (upper block) and 15 offset (lower block) thresholds. For the signal in channel 1 (shown twice as thick black curves on the front sides of the upper and lower blocks), resulting spikes are marked by circles (onset) and squares (offset) with colors indicating respective threshold levels (colorbar). (C) Spikes (onset, top, and offset, bottom) from all 992 afferents plotted as a function of time (*x*-axis) and corresponding frequency channel (*y*-axis). The color of each spike (short thin lines) indicates the threshold level (as used for circles and squares in [B]) of the eliciting unit.

To address time-warp–invariant speech processing, we studied a neuronal module that learns to perform word-recognition tasks. Our model consists of two auditory processing stages. The first stage (Figure 6) consists of an afferent population of neurons that convert incoming acoustic signals into spike patterns by encoding the occurrences of elementary spectrotemporal events. This layer forms a 2-dimensional tonotopy-intensity auditory map. Each of its afferents generates spikes by performing an onset or offset threshold operation on the power of the acoustic signal in a given frequency band. Whereas an onset afferent elicits a spike whenever the log signal power crosses its threshold level from below, offset afferents encode the occurrences of downward crossings (see Materials and Methods) (cf. also [6],[23]). Different on and off neurons coding for the same frequency band differ in their threshold value, reflecting a systematic variation in their intensity tuning. The second, downstream, layer consists of neurons with plastic synaptic peak conductances that are governed by the conductance-based tempotron plasticity rule. These neurons are trained to perform word discrimination tasks. We tested this model on a digit-recognition benchmark task with the TI46 database [24]. We trained each of the 20 conductance-based tempotrons of the second layer to perform a distinct gender-specific binary classification, requiring it to fire in response to utterances of one digit and speaker gender, and to remain quiescent for all other stimuli. After training, the majority of these digit detector neurons (70%) achieved perfect classification of the test set, and the remaining ones performed their task with a low error (Table 1). Based on the spiking activity of this small population of digit detector neurons, a full digit classifier (see Materials and Methods) that weighted spikes according to each detector's individual performance, achieved an overall word error rate of 0.0017. This performance matches the error rates of state-of-the-art artificial speech-recognition systems such as the Hidden Markov model–based Sphinx-4 and HTK, which yield error rates of 0.0017 [25] and 0.0012 [26], respectively, on the same benchmark.

**Table 1:** Test set error fractions of individual detector neurons.

Digit	Male	Female
0	0.0	0.0
1	0.0	0.0
2	0.0008	0.0017
3	0.0	0.0
4	0.0	0.0
5	0.0029	0.0062
6	0.0	0.0
7	0.0004	0.0008
8	0.0	0.0
9	0.0	0.0

### Learned Spectrotemporal Target Features

**Figure 7:**
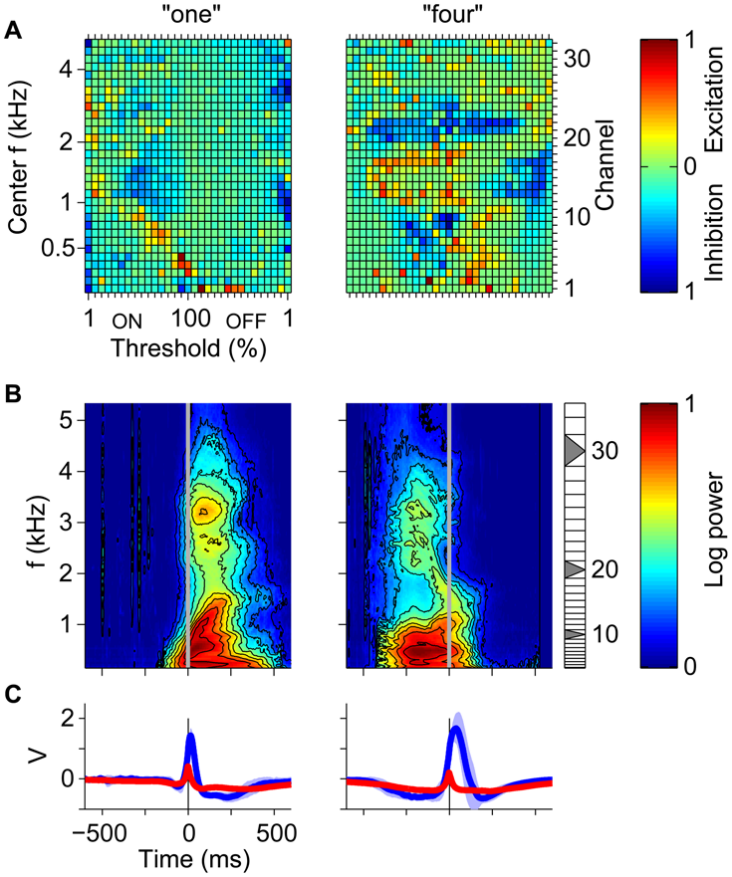
Speech-recognition task. (A) Learned synaptic peak conductances. Each pixel corresponds to one synapse characterized by its frequency channel (right *y*-axis) and its onset (ON) or offset (OFF) afferent power threshold level (*x*-axis, in percent of maximum signal powers [see Materials and Methods]). Learned peak conductances were color coded with excitatory (warm colors) and inhibitory conductances (cool colors) separately normalized to their respective maximal values (color bar). The left *y*-axis shows the logarithmically spaced center frequencies (Mel scale) of the frequency channels. (B) Spike-triggered target stimuli (color-code scaled between the minimum and maximum mean log power). (C) Mean voltage traces for target (blue, light blue ±1 s.d.; spike triggered) and null stimuli (red; maximum triggered).

To reveal qualitatively some of the mechanisms used by our digit detector neurons to selectively detect their target word, we compared the learned synaptic distributions (Figure 7A) of two digit detector neurons (“one” and “four”) to the average spectrograms of each neuron's target stimuli aligned to the times of its output spikes (Figure 7B; see Materials and Methods). The spectrotemporal features that preceeded the output spikes (time zero, grey vertical lines) corresponded to the frequency-specific onset and offset selectivity of the excitatory afferents (Figure 7A, warm colors). These examples demonstrate how the patterned excitatory and inhibitory inputs from both onset and offset neurons are tuned to features of the speech signal. For instance, a prominent feature in the averaged spectrogram of the word “one” (male speakers) was the increase in onset time of the power in the low-frequency channels with the frequency of the channel (Figure 7B, left, channels 1–16). This gradual onset was encoded by a diagonal band of excitatory onset afferents whose thresholds decreased with increasing frequency (Figure 7A, left). By compensating for the temporal lag between the different lower-frequency channels, this arrangement ensured a strong excitatory drive when a target stimulus was presented to the neuron. The spectrotemporal feature learned by the word “four” (male speakers) detector neuron combined decreasing power in the low-frequency range with rising power in the mid-frequency range (Figure 7B, right). This feature was encoded by synaptic efficacies through a combination of excitatory offset afferents in the low-frequency range (Figure 7A, right, channels 1–11) and excitatory onset afferents in the mid-frequency range (channels 12–19). Excitatory synaptic populations were complemented by inhibitory inputs (Figure 7A, blue patches) that prevented spiking in response to null stimuli and also increased the total synaptic conductance. The substantial differences between the mean spike-triggered voltage traces for target stimuli (Figure 7C, blue) and the mean maximum-triggered voltage traces for null stimuli (red) underline the high target word selectivity of the learned synaptic distributions as well as the relatively short temporal extend of the learned target features.

In the examples shown, the average position of the neural decision relative to the target stimuli varied from early to late (Figure 7B, left vs. right). This important degree of freedom stems from the fact that the tempotron decision rule does not constrain the time of the neural decision. As a result, the learning process in each neuron can select the spectrotemporal target features from any time window within the word. The selection of the target feature by the learning takes into account both the requirement of triggering output spikes in response to target stimuli as well as the demand to remain silent during null stimuli. Thus, for each target neuron, the selected features reflect the statistics of both the target and the null stimuli.

### Generalization Abilities of Word Detector Neurons

We have performed several tests designed to assess the ability of the model word detector neurons to perform well on new input sets, different in statistics from the trained database. First, we assessed the ability of the neurons to generalize to unfamiliar speakers and dialects. After training the model with the TI46 database, as described above, we measured its digit-recognition performance on utterances from another database, the TIDIGITS database [27], which includes speech samples from a variety of English dialects (see Materials and Methods). This test has been done without any retraining of the network synapses. The resulting word error rate of 0.0949 compares favorably to the performance of the HTK system, which resulted in an error rate of 0.2156 when subjected to the same generalization test (see Materials and Methods). Across all dialects, our model performed perfectly for roughly one-quarter of all speakers and with at most one error for half of them. Within the best dialect group, an error of at most one word was achieved for as many as 80% of the speakers (Table S1). These results underline the ability of our neuronal word-recognition model to generalize to unfamiliar speakers across a wide range of different unfamiliar dialects.

**Figure 8:**
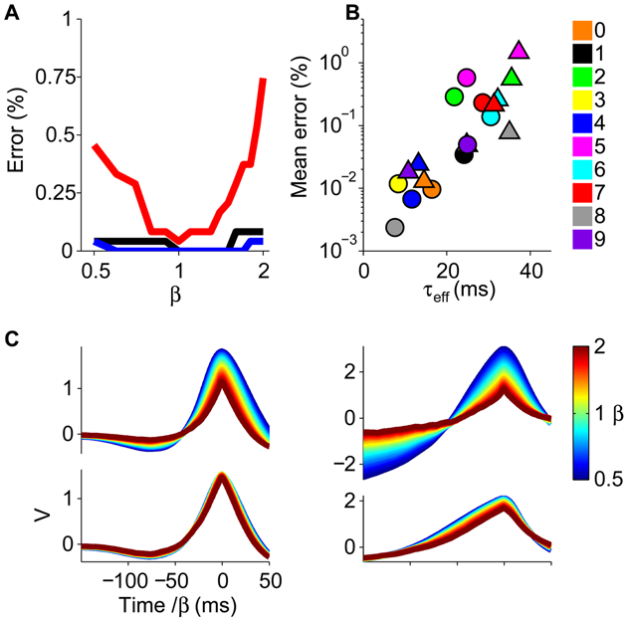
Time-warp robustness. (A) Error versus time-warp factor *β*. (B) Mean errors over the range of *β* shown in (A) (digit color code; triangles: female speakers, circles: male speakers) versus the mean effective time constant *τ*_eff_ calculated for *β* = 1 by averaging the total synaptic conductance over 100-ms time windows prior to either the output spikes (target stimuli) or the voltage maxima (null stimuli). (C) Mean voltage traces for time-warped target patterns for the neurons shown in Figure 7. Bottom row: conductance-based neurons, upper row: current-based neurons (see Materials and Methods).

An interesting question is whether our model neurons are able to generalize their performance to novel time-warped versions of the trained inputs. To address this question, we have tested their performance on randomly generated time-warped versions of the input spikes corresponding to the trained word utterances, without retraining. As shown in Figure 8, the neurons exhibited considerable time-warp–robust performance on the digit-recognition task. For instance, the errors for the “one” (Figure 8A, black line) and “four” (blue line) detector neurons (cf. Figure 7) were insensitive to a 2-fold time warp of the input spike trains. The “seven” detector neuron (male, red line) showed higher sensitivity to such warping; nevertheless, its error rate remained low. Consistent with the proposed role of synaptic conductances, the degree of time-warp robustness was correlated with the total synaptic conductance, here quantified through the mean effective integration time *τ*_eff_ (Figure 8B). Additionally, the mean voltage traces induced by the target stimuli (Figure 8C, lower traces) showed a substantially smaller sensitivity to temporal warping than their current-based analogs (see Materials and Methods) (Figure 8C, upper traces).

**Figure 9:**
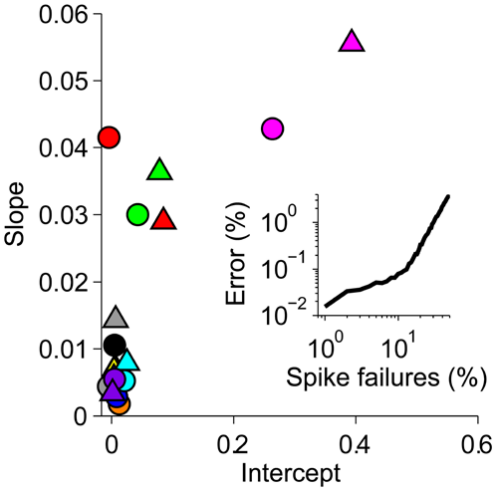
Robustness to spike failures. The error fraction of each digit detector neuron was measured as a function of the spike failure probability over the range from 0% to 10% and fitted by linear regression. For each neuron, the resulting slope (median 0.0069) is plotted versus the intercept (median 0.0061) with symbols and colors as in Figure 8B. The median *R* ^2^ of the linear regression fits was 0.94. The inset shows the median error fraction of the population as a function of the spike failure probability in the range of 1% to 50% with the robust regime braking down at approximately 20%.

We also found that our model word detector neurons are robust to the introduction of spike failures in their input patterns. For each neuron, we have measured its performance on inputs which were corrupted by randomly deleting a fraction of the incoming spikes, again without retraining. For the majority of neurons, the error percentage increased by less than 0.01% for each percent increase in spike failures (Figure 9). This high robustness reflects the fact that each classification is based on integrating information from many presynaptic sources.

## Discussion

### Automatic Rescaling of Effective Integration Time by Synaptic Conductances

The proposed conductance-based time-rescaling mechanism is based on the biophysical property of neurons that their effective integration time is shaped by synaptic conductances and therefore can be modulated by the firing rate of its afferents. To utilize these modulations for time-warp–invariant processing, a central requirement is a large evoked total synaptic conductance that dominates the effective integration time constant of the postsynaptic cell through shunting. In our speech-processing model, large synaptic conductances with a median value of a 3-fold leak conductance across all digit detector neurons (cf. Figure 8B) result from a combination of excitatory and inhibitory inputs. This is consistent with high total synaptic conductances, comprising excitation and inhibition, that have been observed in several regions of cortex [28] including auditory [29],[30], visual [31],[32], and also prefrontal [33],[34] (but see ref. [35]). Our model predicts that in cortical sensory areas, the time-rescaled intracellular voltage traces (cf. Figure 3C), and consequently, also the rescaled spiking responses of neurons that operate in the proposed fashion, remain invariant under temporal warping of the neurons' input spike patterns. These predictions can be tested by intra- and extracellular recordings of neuronal responses to temporally warped sensory stimuli.

A large total synaptic conductance is associated with a substantial reduction in a neuron's effective integration time relative to its resting value. Therefore, the resting membrane time constant of a neuron that implements the automatic time-rescaling mechanism must substantially exceed the temporal resolution that is required by a given processing task. Because the word-recognition benchmark task used here comprises whole-word stimuli that favored effective time constants on the order of several tens of milliseconds, we used a resting membrane time constant of *τ*_m_ = 100 ms. Whereas values of this order have been reported in hippocampus [36] and cerebellum [21],[37], it exceeds current estimates for neocortical neurons, which range between 10 and 30 ms [35],[38],[39]. Note, however, that the correspondence of our passive membrane model and the experimental values that typically include contributions from various voltage-dependent conductances is not straightforward. Our model predicts that neurons specialized for time-warp–invariant processing at the whole-word level have relatively long resting membrane time constants. It is likely that the auditory system solves the problem of time-warp–invariant processing of the sound signal primarily at the level of shorter speech segments such as phonemes. This is supported by evidence that primary auditory cortex has a special role in speech processing at a resolution of milliseconds to tens of milliseconds [11]–[13]. Our mechanism would enable time-warp–invariant processing of phonetic segments with resting membrane time constants in the range of tens of milliseconds, and much shorter effective integration times.

The proposed neuronal time-rescaling mechanism assumes linear summation of synaptic conductances. This assumption is challenged by the presence of voltage-dependent conductances in neuronal membranes. Since the potential implications for our model depend on the specific nonlinearity induced by a cell-type–specific composition of different ionic channels, it is hard to evaluate the overall effect on our model in general terms. Nevertheless, because of its immanence, we expect the conductance-based time-rescaling mechanism to cope gracefully with moderate levels of nonlinearity. As an example, we tested its behavior in the presence of an h-like conductance (see Materials and Methods) that opposes conductance changes induced by depolarizing excitatory synaptic inputs and is active at the resting potential. As expected, we found that physiological levels of h-conductances resulted in only moderate impairment of the automatic time-rescaling mechanism (Figure S1).

For the sake of simplicity as well as numerical efficiency, we have assumed symmetric roles of excitation and inhibition in our model architecture. We have checked that this assumption is not crucial for the operation of the automatic time-rescaling mechanism and the learning of time-warped random latency patterns. Specifically, we have implemented the random latency classification task for a control architecture in which all synapses were confined to be excitatory except a single global inhibitory input that, mimicking a global inhibitory network, received a separate copy of all incoming spikes. In this architecture, all spike patterns have to be encoded by the excitatory synaptic population, and the role of inhibition is reduced to a global signal that has equal strength for all input patterns. Due to the limitations of this architecture, this model showed some reduction of storage capacity relative to the symmetric case, but the automatic time-rescaling mechanism remained intact. For a time-warp scale of *β*_max_ = 2.5 (cf. Figure 3), the global inhibition model roughly matched the performance of the symmetric model when the learning load was lowered to 1.5 spike patterns per synapse, with an error fraction of 0.18%.

### Supervised Learning of Synaptic Conductances

To utilize synaptic conductances as efficient controls of the neuron's clock, the peak synaptic conductances must be plastic so that they adjust to the range of integration times relevant for a given perceptual task. This was achieved in our model by our novel supervised spike-based learning rule. This plasticity posits that the temporal window during which pre- and postsynaptic activity interact continuously adapts to the effective integration time of the postsynaptic cell (Figure 4). The polarity of synaptic changes is determined by a supervisory signal that we hypothesize to be realized through neuromodulatory control [22]. Because present experimental measurements of spike-timing–dependent synaptic plasticity rules have assumed an unsupervised setting, i.e., have not controlled for neuromodulatory signals (but see [40]), existing results do not directly apply to our model. Nevertheless, recent data have revealed complex interactions between the statistics of pre- and postsynaptic spiking activity and the expression of synaptic changes [41]–[44]. Our model offers a novel computational rationale for such interactions, predicting that for fixed supervisory signaling, the temporal window of plasticity shrinks with growing levels of postsynaptic shunting. One challenge for the biological implementation of the tempotron learning rule is the need to compute the time of the maximum of the postsynaptic voltage. We have previously shown for a current-based neuron model that this temporally global operation can be approximated by temporally local computations that are based on the postsynaptic voltage traces following input spikes [22]. We have extended this approach to plastic synaptic conductances and checked that the resulting biologically plausible implementation of conductance-based tempotron learning can readily subserve time-warp–invariant classification of spike patterns. Specifically, in this implementation, the induction of synaptic plasticity is controled by the correlation of the postsynaptic voltage and a synaptic learning kernel (see Materials and Methods) whose temporal extend is controlled by the average conductance throughout a given error trial. A synaptic peak conductance is changed by a uniform amount whenever this correlation exceeds a fixed plasticity induction threshold. When tested on the time-warped latency patterns with *β*_max_ = 2.5 (cf. Figure 3), the correlation-based tempotron roughly matched the voltage maximum–based version at a reduced learning load of 1.5 patterns per synapse with an error fractions of 0.35%.

### Time-Warp Invariance Is Task Dependent

In our model, dynamic time-warp–invariant capabilities become avaliable through a conductance-based learning rule that tunes the shunting action of synaptic conductances. This learning rule enables neurons to adjust the degree of synaptic shunting to the requirements of a given processing task. As a result, our model can naturally encompass a continuum of functional specializations ranging from neurons that are sensitive to absolute stimulus durations by employing low total synaptic conductances, to time-warp–invariant feature detectors that operate in a high-conductance regime. In the context of auditory processing, such a functional segregation into neurons with slower and faster effective integration times is reminiscent of reports suggesting that rapid temporal processing in time frames of tens of milliseconds is localized in left lateralized language areas, whereas processing of slower temporal features is attributed to right hemispheric areas [45]–[47]. Although anatomical and morphological asymmetries between left and right human auditory cortices are well documented [48], it remains to be seen whether these differences form the physiological substrate for a left lateralized implementation of the proposed time-rescaling mechanism. Consistent with this picture, the general tradeoff between high temporal resolution and robustness to temporal jitter that is predicted by our model (Figure 5) parallels reports of the vulnerability of the lateralizion of language processing with respect to background acoustic noise [49] as well as to abnormal timing of auditory brainstem responses [50].

### Neuronal Circuitry for Time-Warp–Invariant Feature Detection

The architecture of our speech-processing model encompasses two auditory processing stages. The first stage transforms acoustic signals into spatiotemporal patterns of spikes. To engage the proposed automatic time-rescaling mechanism, the population rate of spikes elicited in this afferent layer must track variations in the rate of incoming speech. Such behavior emerges naturally in a sparse coding scheme in which each neuron responds transiently to the occurrences of a specific acoustic event within the auditory input. As a result, variations in the rate of acoustic events are directly translated into concomitant variations in the population rate of elicited spikes. In our model, the elementary acoustic events correspond to onset and offset threshold crossings of signal power within specific frequency channels. Such frequency-tuned onset and offset responses featuring a wide range of dynamic thresholds have been observed in the inferior colliculus (IC) of the auditory midbrain [51]. This nucleus is the site of convergence of projections from the majority of lower auditory nuclei and is often referred to as the interface between the lower brain stem auditory pathways and the auditory cortex. Correspondingly, we hypothesize that the layer of time-warp–invariant feature detector neurons in our model implements neurons located downstream of the IC, most probably in primary auditory cortex. Current studies on the functional role of the auditory periphery in speech perception and its pathologies have been limited by the lack of biologically plausible neuronal readout architectures; a limitation overcome by our model, which allows evaluation of specific components of the auditory pathway in a functional context.

### Implications for Speech Processing

Psychoacoustic studies have indicated that the neural mechanism underlying the perceptual normalization of temporal speech cues is involuntary, i.e., it is cognitively impenetrable [16], controlled by physical rather than perceived speaking rate [17], confined to a temporally local context [2],[18], not specific to speech sounds [52], and already operational in prearticulate infants [53]. The proposed conductance-based time-rescaling mechanism is consistent with these constraints. Moreover, our model posits a direct functional relation between high synaptic conductances and the time-warp robustness of human speech perception. This relation gives rise to a novel mechanistic hypothesis explaining the impaired capabilities of elderly listeners to process time-compressed speech [54],[55]. We hypothesize that the down-regulation of inhibitory neurotransmitter systems in aging mammalian auditory pathways [56],[57] limits the total synaptic conductance and therefore prevents the time-rescaling mechanism from generating short, effective time constants through synaptic shunting. Furthermore, our model implies that comprehension deficits in older adults should be linked specifically to the processing of phonetic segments that contain fast time-compressed temporal cues. Our hypothesis is consistent with two interrelated lines of evidence. First, comprehension difficulties of time-compressed speech in older adults are more likely a consequence of an age-related decline in central auditory processing than attributes of a general cognitive slowing [56],[58]. Second, recent reports have indicated that recognition differences between young and elderly listeners originate mainly from the temporal compression of consonants, which often feature rapid spectral transitions, but not from steady-state segments [54],[55],[58] of speech. Finally, our hypothesis posits that speaking rate–induced shifts in perceptual category boundaries [2],[16],[17] should be age-dependent, i.e., their magnitude should decrease with increasing listener age. This prediction is straightforwardly testable within established psychoacoustic paradigms.

### Connections to Other Models of Time-Warp–Invariant Processing

In a previous neuronal model of time-warp–invariant speech processing [5],[6], sequences of acoustic events are converted into patterns of transiently matching firing rates in subsets of neurons within a population, which trigger synchronous firing in a downstream readout circuit. The identity of neurons whose firing rates converge to an identical value during an input pattern, and hence also the pattern of synchrony emerging in the readout layer, depends only on the relative timing of the events, not on the absolute duration of the auditory signal. However, for this model to recognize multiple input patterns, the convergence of firing rates is only approximate. Therefore, the resulting time-warp robustness is limited and, as in our model, dependent on the learning load. Testing this model on our synthetic classification task (cf. Figure 3) indicated a substantially smaller storage capacity than is realizable by the conductance-based tempotron (Text S1). An additional disadvantage of this approach is that it copes only with global (uniform) temporal warping. Invariant processing of dynamic time warp as is exhibited by natural speech (cf. Figure 1C and 1D) is more challenging since it requires robustness to local temporal distortions of a certain statistical character. Established algorithms that can cope with dynamically time-warped signals are typically based on minimizing the deviation between an observed signal and a stored reference template [59]–[61]. These algorithms are computationally expensive and lack biologically plausible neuronal implementations. By contrast, our conductance-based time-rescaling mechanism results naturally from the biophysical properties of input integration at the neuronal membrane and does not require dedicated computational resources. Importantly, our model does not rely on a comparison between the incoming signal and a stored reference template. Rather, after synaptic conductances have adjusted to the statistics of a given stimulus ensemble, the mechanism generalizes and automatically stabilizes neuronal voltage responses against dynamic time warp even when processing novel stimuli (cf. Figure 3C). The architecture of our neuronal model also fundamentally departs from the decades-old layout of Hidden Markov Model–based artificial speech-recognition systems, which employ probabilistic models of state sequences. These systems are hard to reconcile with the biological reality of neuronal system architecture, dynamics, and plasticity. The similarity in performance between our model and such state-of-the-art systems on a small vocabulary task highlights the powerful processing capabilities of spike-based neural representations and computation.

### Generality of Mechanism

Although the present work focuses on the concrete and well-documented example of time-warp robustness in the context of neural speech processing, the proposed mechanism of automatic rescaling of integration time is general and applies also to other problems of neuronal temporal processing such as birdsong recognition [3], insect communication [9], and other ethologically important natural auditory signals. Moreover, robustness of neuronal processing to temporal distortions of spike patterns is not only important for the processing of stimulus time dependencies, but also in the context of spike-timing–based neuronal codes in which the precise temporal structure of spiking activity encodes information about nontemporal physical stimulus dimensions [62]. Evidence for such temporal neural codes have been reported in the visual [63]–[65], auditory [66], and somatosensory [67], as well as the olfactory [68] pathways. As a result, we expect mechanisms of time-warp–invariant processing to also play a role in generating perceptual constancies along nontemporal stimulus dimensions such as contrast invariance in vision or concentration invariance in olfaction [4]. Finally, time warp has also been described in intrinsically generated brain signals. Specifically, the replay of hippocampal and cortical spiking activity at variable temporal warping [69],[70] suggests that our model has applicability beyond sensory processing, possibly also encompassing memory storage and retrieval.

## Materials and Methods

### Conductance-Based Neuron Model

Numerical simulations of the conductance-based tempotron were based on exact integration [71] of the conductance-based voltage dynamics defined in Equation 1. With the membrane capacitance set to 1, the resting membrane time constant in this model is *τ*_m_ = 1/*g*_leak_. Implementing an integrate-and-fire neuron model, an output spike was elicited when *V*(*t*) crossed the firing threshold *V*_thr_. After a spike at *t*_spike_, the voltage is smoothly reset to the resting value by shunting all synaptic inputs that arrive after *t*_spike_ (cf. [22]). We used *V*_thr_ = 1, *V*_rest_ = 0, and reversal potentials 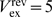 and 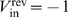 for excitatory and inhibitory conductances, respectively. Unless stated otherwise, the resting membrane time constant was set to *τ*_m_ = 100 ms throughout our work [20]. For the synaptic time constant, we used *τ*_s_ = 1 ms for the random latency task, which minimized the error of the current-based neuron, and to *τ*_s_ = 5 ms in the speech-recognition tasks.

### H-Current

To check the effect of nonsynaptic voltage-dependent conductances on the automatic time-rescaling mechanism, we implemented an h-like current *I*_h_ after [72] as a noninactivating current with HH-type dynamics of the form
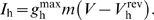



Here, 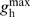 is the maximal h-conductance, with reversal potential 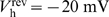 and *m* is its voltage-dependent activation variable with kinetics
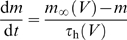
where
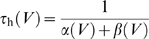
and
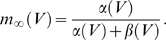



The voltage dependence of the rate constants *α* and *β* were described by the form
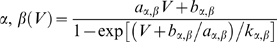
with parameters *a*_α_ = −39.015 s^−1^, *b*_α_ = −259.925 s^−1^, *k*_α_ = 1.77926 and *a*_β_ = 365.85 s^−1^, *b*_β_ = −2853.25 s^−1^, *k*_β_ = −1.28889.

In Figure S1, we quantified the effect of the h-conductance on the fidelity of the time-rescaling mechanism by measuring the time-warp–induced distortions of neuronal voltage traces for different values of the maximal h-conductance 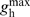. Specifically, for a given value of 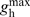 and a time warp *β*, we measure the voltage traces 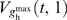 and 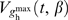 and their standard deviations across time *σ*_1_ and *σ_β_*, respectively. We define the time-warp distortion index 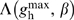 as the mean magnitude of the time-warp–induced voltage difference across time normalized by the mean standard deviation, 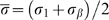,
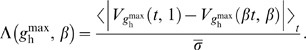



In Figure S1, values of 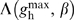 are normalized by Λ(0,*β*). The voltage traces were generated by random latency patterns and uniform synaptic peak conductances as used in Figure 2. As increasing values of 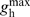 depolarized the neuron's resting potential, excitatory and inhibitory synaptic conductances were balanced separately for each value of 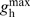.

### Current-Based Neuron Model

In the current-based tempotron that was implemented as described in [22], each input spike evoked an exponentially decaying synaptic current that gave rise to a postsynaptic potential with a fixed temporal profile. In Figure 8C (upper row), voltage traces of a current-based analog of a conductance-based tempotron with learned synaptic conductances 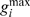, reversal potentials 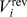, and effective membrane integration time *τ*_eff_ (cf. Figure 8B) were computed by setting the synaptic efficacies *ω_i_* of the current-based neuron to 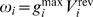 and its membrane time constant to *τ*_m_ = *τ*_eff_. The resulting current-based voltage traces were scaled such that for each pair of models, the mean voltage maxima for unwarped stimuli (*β* = 1) were equal.

### Tempotron Learning

Following [22], changes in the synaptic peak conductance 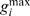 of the *i*th synapse after an error trial were given by the gradient of the postsynaptic potential, 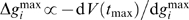, at the time of its maximal value *t*_max_. To compute the synaptic update for a given error trial, the exact solution of Equation 1 was differentiated with respect to 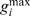 and evaluated at *t*_max_, which was determined numerically for each error trial. Whenever a synaptic peak conductance attempted to cross to a negative value, its reversal potential was switched.

### Voltage Correlation-Based Learning

A voltage correlation-based approximation of tempotron learning was implemented by extending the approach in [22] such that the change in the synaptic peak conductance 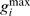 of the *i*th synapse due to a spike at time *t_i_* was governed by the correlation 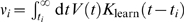 of the postsynaptic potential *V*(*t*) with a synaptic learning kernel *K*_learn_(*t*) = (exp(−*t/τ*_learn_)−exp(−*t/τ*_s_))/(*τ*_learn_−*τ*_s_). The two time constants of the synaptic learning kernel were the synaptic time constant *τ*_s_ and the learning time constant 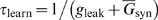, which was determined by the time-averaged synaptic conductance 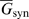 of the current error trial and approximated the effective membrane time constant during that trial. The voltage maximum operation was approximated by thresholding *ν_i_*, yielding
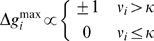
for changes of excitatory conductances on target and null patterns, respectively, and changes with the reversed polarity, ±1, for inhibitory conductances. The plasticity induction threshold was set to *κ* = 0.45.

### Learning Rate and Momentum Term

As in [22], we employed a momentum heuristic to accelerate learning in all learning rules. In this scheme, synaptic updates 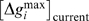 consisted, not only of the correction 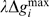, which was given by the learning rule and the learning rate *λ*, but also incorporated a fraction *μ* of the previous synaptic change 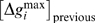. Hence, 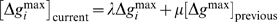. We used an adaptive learning rate that decreased from its initial value *λ*_ini_ as the number of learning cycles *l* grew, *λ* = *λ*_ini_/(1+10^−4^(*l*−1)). A learning cycle corresponded to one iteration through the batch of templates in the random latency task or the training set in the speech task.

#### Random latency task training

To ensure a fair comparison between the conductance-based and the current-based tempotrons (cf. Figure 3A), the learning rule parameters *λ*_ini_ and *μ* were optimized for each model. Specifically, for each value of *β*_max_, optimal values over a 2-dimensional grid were determined by the minimal error frequency achieved during runs over 10^5^ cycles, with synaptic efficacies starting from Gaussian distributions with zero mean and standard deviations of 0.001. The optimization was performed over five realizations.

### Global Time Warp

Global time warp was implemented by multiplying all firing times of a spike template by a constant scaling factor *β*. In Figure 3A, random global time warp between compression by 1/*β*_max_ and dilation by *β*_max_ was generated by setting *β* = exp(*q*ln(*β*_max_)) with *q* drawn from a uniform distribution between −1 and 1 for each presentation.

### Dynamic Time Warp

Dynamic time warp was implemented by scaling successive interspike intervals *t_j_*−*t_j−1_* of a given template with a time-dependent warping factor 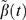, such that warped spike times 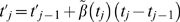 with 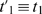 and 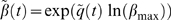. The time-dependent factor 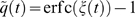 resulted from an equilibrated Ornstein-Uhlenbeck process *ξ*(*t*) with a relaxation time of *τ* = 200 ms that was rescaled by the complementary error function erfc to transform the normal distribution of *ξ*(*t*) into a uniform distribution over [−1 1] at each *t*.

### Global Inhibition Model

To ensure that the symmetry of excitation and inhibition in our model architecture was not crucial for the time-warp–invariant processing of spike patterns, we implemented a control architecture in which all afferents were confined to be excitatory, except one additional inhibitory synapse, which mimicked the effect of a global inhibitory network. In the time-warped random latency task, spike patterns were fed into the excitatory population as before; however, in addition, the inhibitory synapse received a copy of each incoming spike. All synaptic peak conductances were plastic and controlled by the conductance-based tempotron rule. In this model, synaptic sign changes were prohibited.

### Gaussian Spike Time Jitter

Spike time jitter [22] was implemented by adding independent Gaussian noise with zero mean and a standard deviation of 5 ms to each spike of a template before each presentation.

### Acoustic Front-End

Sound signals were normalized to unit peak amplitude and converted into spectrograms over *N*_FTT_ = 129 linearly spaced frequencies *f_j_* = *f*_min_+*j*(*f*_max_+*f*_min_)/(*N*_FTT_+1) (*j* = 1… *N*_FTT_) between *f*_min_ = 130 Hz and *f*_max_ = 5,400 Hz by a sliding fast Fourier transform with a window size of 256 samples and a temporal step size of 1 ms. The resulting spectrograms were filtered into *N*_f_ = 32 logarithmically spaced Mel frequency channels by overlapping triangular frequency kernels. Specifically, *N*_f_+2 linearly spaced frequencies given by *h_j_* = *h*_min_+*j*(*h*_max_−*h*_min_)/(*N*_f_+1) with *j* = 0…*N*_f_+1 and *h*_max,min_ = 2,595log(1+*f*_max,min_/700) were transformed to a Mel frequency scale 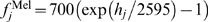 between *f*_min_ and *f*_max_. Based on these, signals in *N*_f_ channels resulted from triangular frequency filters over intervals 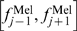 with center peaks at 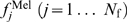. After normalization of the resulting Mel spectrogram *S* ^Mel^ to unit peak amplitude, the logarithm was taken through log(*S* ^Mel^ = ε)−log(ε) with ε = 10^−5^ and the signal in each frequency channel smoothed in time by a Gaussian kernel with a time constant of 10 ms. Spikes were generated by thresholding of the resulting signals by a total of 31 onset and offset threshold-crossing detector units. Whereas each onset afferent emitted a spike whenever the signal crossed its threshold in the upward direction, offset afferents fired when the signal dropped below the threshold from above. For each frequency channel and each utterance, threshold levels for onset and offset afferents were set relative to the maximum signal over time to 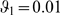 and 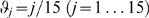. For 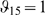, onset and offset afferents were reduced to a single afferent whose spikes encoded the time of the maximum signal for a given frequency channel.

### Speech Databases

We used the digit subset of the TI46 Word speech database [24]. This clear speech dataset comprises 26 isolated utterances of each English digit from zero to nine spoken by 16 adult speakers (eight male and eight female). The data is partitioned into a fixed training set, comprising 10 utterances per digit and speaker, and a fixed test set holding the remaining 16 utterances per digit and speaker. We also tested our neuronal word-recognition model on the adult speaker, isolated-digit subset of the TIDIGITS database [27]. This subset comprises two utterances per digit and speaker, i.e., a total of 20 utterances from 225 adult speakers (111 male and 114 female), that are dialectically balanced across 21 dialectical regions (tiling the continental United States). Because the TI46 database only provides utterances of the word “zero” for the digit 0, we excluded the utterances of “oh” from our TIDIGITS sample.

### Digit Classification

Based on the spiking activity of all binary digit detector neurons, a full digit classifier was implemented by ranking the digit detectors according to their individual task performances. As a result, a given stimulus was classified as the target digit of the most reliable of all responding digit detector neurons. If all neurons remained silent, a stimulus was classified as the target digit of the least reliable neuron.

### Spike-Triggered Target Features

To preserve the timing relations between the learned spectrotemporal features and the target words, we refrained from correcting the spike-triggered stimuli for stimulus autocorrelations [73].

### Speech Task Training

Test errors in the speech tasks were substantially reduced by training with a Gaussian spike jitter with a standard deviation of σ added to the input spikes as well a symmetric threshold margin *v* that required the maximum postsynaptic voltage on target stimuli to exceed *V*_thr_+*v* and to remain below *V*_thr_−*v* during null stimuli. Values of *λ*_ini_, *μ*, *σ*, and *v* were optimized on a 4-dimensional grid. Because for each grid point, only short runs over maximally 200 cycles were performed, we also varied the mean values of initial Gaussian distributions of the excitatory and inhibitory synaptic peak conductances, keeping their standard deviations fixed at 0.001. The reported performances are based on the solutions that had the smallest errors fractions over the test set. If not unique, we selected the solution with the highest robustness to time warp (cf. Figure 8B). Note that this naive optimization of the training parameters did not maintain a separate holdout test set for cross-validation and might therefore overestimate the true generalization performance. We adopted this optimization scheme from [25],[26] to ensure comparability of the resulting performance measures.

### Comparison to the HTK

HTK generalization performance was tested with the HTK package version 3.4.1 [74] with front-end and HMM model parameters following [26]. Specifically, speech data from the TI46 and TIDIGITS databases were converted to 13 Mel-cepstral coefficients (including the 0*th* order coefficient) along with their first and second derivatives at a frame rate of 5 ms. Mel-coefficients were computed over 30 channels in 25-ms windows with zero mean normalization enabled (TARGETKIND = MFCC_D_A_Z_0). In addition, the following parameters were set: USEHAMMING = T; PREEMPCOEF = 0.97; and CEPLIFTER = 22. Ten HMM models, one for each digit plus one HMM model for silence, were used. Each model consisted of five states (including the the two terminal states) with eight Gaussian mixtures per state and left-to-right (no skip) transition topology.

## Supporting Information

Figure S1
**Effect of h-conductance on time rescaling.** Time-warp distortion index computed for random latency patterns (see Materials and Methods) versus the maximal h-conductance for different values of the mean synaptic conductance 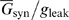: 7.2 (triangles), 10.8 (squares), and 14.4 (circles). Curves were averaged over 2,000 spike-pattern realizations.(0.70 MB TIF)

Table S1 **Generalization from TI46 to TIDIGITS.** For each dialect group, the table lists the percentages of speakers for which our model committed a given number of word-recognition errors.
(0.01 MB PDF)

Text S1 **Comparison to the Hopfield-Brody model of time-warp–invariant neuronal processing.**
(0.03 MB PDF)
