# Supplementary figures and images for: Time-Warp–Invariant Neuronal Processing
Source: PLoS Biol. 2009 Jul 7;7(7):e1000141. doi: 10.1371/journal.pbio.1000141 (PMC2701607; doi:10.1371/journal.pbio.1000141)

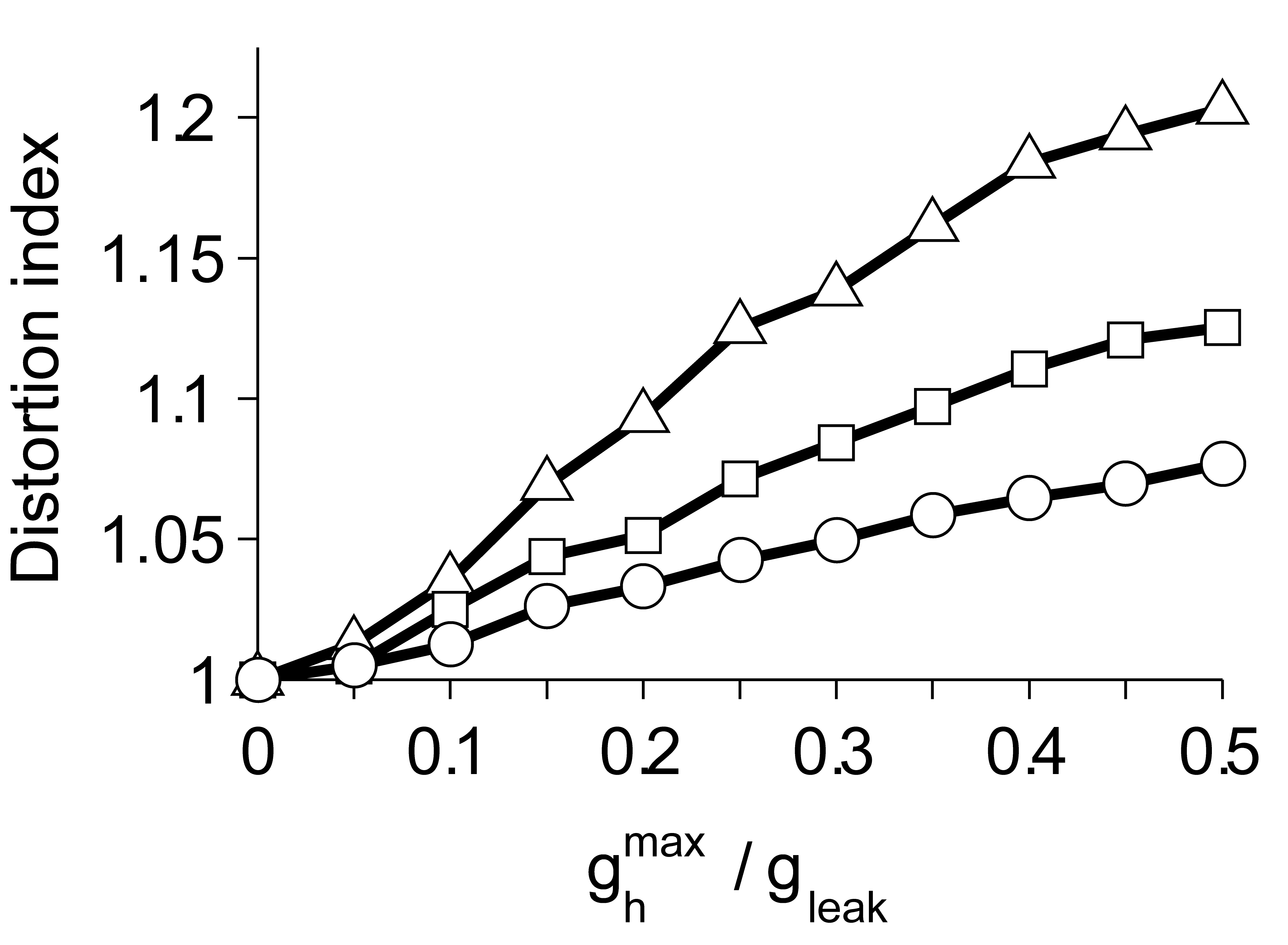

Supplement: Figure S1 — Effect of h-conductance on time rescaling. Time-warp distortion index computed for random latency patterns (see Materials and Methods) versus the maximal h-conductance for different values of the mean synaptic conductance : 7.2 (triangles), 10.8 (squares), and 14.4 (circles). Curves were averaged over 2,000 spike-pattern realizations. (0.70 MB TIF) [file pbio.1000141.s001.tif]
