# Supplementary material for: Time-Warp–Invariant Neuronal Processing
Source: PLoS Biol. 2009 Jul 7;7(7):e1000141. doi: 10.1371/journal.pbio.1000141 (PMC2701607; doi:10.1371/journal.pbio.1000141)
Supplement: Table S1 — Generalization from TI46 to TIDIGITS. For each dialect group, the table lists the percentages of speakers for which our model committed a given number of word-recognition errors. (0.01 MB PDF) [file pbio.1000141.s002.pdf]

Number of errors	0	1	2	3	4	5	6	7	8	9	10
Overall	23.11	27.11	18.67	12.89	11.56	3.11	1.78	0.44	0.89	0.44	0
Eastern New England	30	20	20	0	20	10	0	0	0	0	0
Virginia Piedmont	30	40	10	20	0	0	0	0	0	0	0
Southwest	40	30	20	10	0	0	0	0	0	0	0
Southern California	40	0	20	20	10	0	10	0	0	0	0
South Midland	10	30	20	20	20	0	0	0	0	0	0
Central New York	33.33	33.33	8.33	16.67	0	0	8.33	0	0	0	0
Rocky Mountains	40	40	20	0	0	0	0	0	0	0	0
North Central	30	40	20	10	0	0	0	0	0	0	0
Delaware Valley	27.27	27.27	18.18	18.18	0	0	0	0	9.09	0	0
Midland	10	30	10	30	10	10	0	0	0	0	0
North Central	20	30	30	10	10	0	0	0	0	0	0
South Carolina	10	30	20	10	10	10	0	10	0	0	0
Gulf South	30	10	20	20	0	10	10	0	0	0	0
South Midland	10	40	40	0	0	10	0	0	0	0	0
Gulf South	10	10	20	10	30	10	0	0	0	10	0
Spanish American	20	40	20	0	20	0	0	0	0	0	0
Southwest	40	10	20	10	10	0	10	0	0	0	0
New York City	0	30	10	20	30	0	0	0	10	0	0
South Midland	36.36	45.45	0	18.18	0	0	0	0	0	0	0
Pacific Northwest	30	30	20	20	0	0	0	0	0	0	0
Upper Ohio Valley	0	30	10	10	50	0	0	0	0	0	0
African American	9.09	0	36.36	9.09	36.36	9.09	0	0	0	0	0

Table S1: Generalization from TI46 to TIDIGITS. For each dialect group the table lists the percentages of speakers for which our model committed a given number of word recognition errors.
