# Supplementary material for: Time-Warp–Invariant Neuronal Processing
Source: PLoS Biol. 2009 Jul 7;7(7):e1000141. doi: 10.1371/journal.pbio.1000141 (PMC2701607; doi:10.1371/journal.pbio.1000141)
Supplement: Text S1 — Comparison to the Hopfield-Brody model of time-warp–invariant neuronal processing. (0.03 MB PDF) [file pbio.1000141.s003.pdf]

Comparison to the Hopfield-Brody model of time-warp invariant neuronal processing.

We implemented a system based on the Hopfield-Brody model [1, 2] of time warp invariant processing, that performs classification of latency patterns, similar to the synthetic task studied in this paper (Figure 3). The system consists of N neurons, each of which responds to an incoming event at time t_i by a linearly decaying firing rate ramp

$$r_i(t) = \left[1 - \frac{(t - t_i)}{\tau_i}\right]_+, \quad t_i \leq t \quad (i = 1, \ldots, N)$$

with the neuron specific decay time τ_i. The rectification $[\,]_+$ limits $r_i(t)$ to non zero values. As in our synthetic latency pattern classification task we considered input patterns that consisted of one event per neuron. Each such pattern had to be classified as target or null. The system classifies a pattern as target if a sufficiently large number of neurons fire with roughly equal rates ("Many-are-Equal"), i.e. their differences are not greater in magnitude than some tolerance interval Δ. In the original implementation in ref.[1, 2] this condition ensured the transient synchronization of cells within a subsequent processing layer. For a given time t, let us denote the number of neurons whose firing rates $r_i(t)$ are equal to r with a tolerance Δ by $C(t, r)$, i.e.

$$C(t, r) = \sum_{i=1}^{N} \chi(r_i(t) - r)$$

where $\chi(x) = 1$ iff $|x| < \Delta$ and 0 otherwise. In order to implement the task only a subset of the inputs are read by the decoding system. We thus define the score

$$S(t, r) = \sum_{i=1}^{N} w_i \chi(r_i(t) - r)$$

where $\{w_i\}$ $(i = 1, \ldots, N)$ denotes a vector of N plastic binary readout weights ($w_i \in \{0, 1\}$). A given input pattern (specifying a vector of t_i's) is classified by the system as target if $\max_{t,r} S(t, r) > \vartheta$ and as null otherwise. The parameter ϑ corresponds to the threshold for synchrony in the original model. For our classification task, where on average half of the patterns are targets, it is reasonable to choose: $\vartheta = \langle \max_{t,r} C(t, r)\rangle/2$ with the average taken over the set of all input patterns.

To test the capacity of this model we introduced a simple heuristic to train the readout weights $\{w_i\}$ to classify a given realization of p input patterns. Starting with a random weight vector with mean one half, patterns are cycled through the circuit. Whenever a null pattern is classified as target pattern, one of the weights contributing to the maximal score is changed from 1 to 0. Conversely, when a target pattern is classified as null, the maximum score $S(t, r)$ for which the corresponding count $C(t, r)$ is larger than the score, is increased by switching one of the vanishing weights to 1. For both types of corrections, preference is given to the candidate weight with the smallest number of total changes.

We have simulated the above model with $N = 800$ neurons whose decay times τ_i were chosen randomly from a uniform distribution between 0.3 s and 1.1 s. As in our synthetic latency pattern classification task we considered input patterns that consisted of one event per neuron arriving at a time that was randomly chosen from a uniform distribution between 0 and $T = 0.5$ s. Each of p input patterns was randomly labeled as target or null pattern with probability one half. The tolerance was $\Delta = 2.5\ 10^{-3}$ s. The numerical results quoted below were not sensitive to modest changes in these parameters.

Without temporal warping of the input patterns, the above learning heuristic successfully stored up to 50 (but not 60) patterns in the proposed architecture, which is in the range of the capacity found in ref. [2], where 25 is cited as the maximum number of patterns successfully recognized. This capacity of $50/800 = 0.0625$ patterns per modifiable synapse is substantially smaller than the storage capacity of the tempotron, which is approximately 3. In addition, we found that near capacity the 'Many Are Equal' model described above is highly sensitive to global temporal warping of the patterns. Requiring that the system will be able to learn to classify correctly time warped latency patterns up to a warping factor of 2, reduces the capacity to well below 25 patterns, suggesting (cf. Figure 3) that the conductance based tempotron has a substantially larger capacity to learn to classify latency patterns in a time warped robust manner. It should be noted that this comparison is limited to the binary weight learning rule described above which is in the spirit of the learning rule suggested in ref. [2]. We cannot rule out the existence of more powerful learning rules that will exhibit a larger capacity for the 'Many Are Equal' model.

1. Hopfield JJ, Brody CD (2000) What is a moment? "cortical" sensory integration over a brief interval. Proc Natl Acad Sci USA 97:13919–131924.

2. Hopfield JJ, Brody CD (2001) What is a moment? transient synchrony as a collective mechanism for spatiotemporal integration. Proc Natl Acad Sci USA 98:1282–1287.
